# Methylphenidate and the risk of psychosis in adolescents and young adults: a population-based cohort study

**DOI:** 10.1016/S2215-0366(19)30189-0

**Published:** 2019-08

**Authors:** Chris Hollis, Qi Chen, Zheng Chang, Patrick D Quinn, Alexander Viktorin, Paul Lichtenstein, Brian D'Onofrio, Mikael Landén, Henrik Larsson

**Affiliations:** a National Institute of Health Research (NIHR) MindTech MedTech Cooperative, NIHR Nottingham Biomedical Research Centre and Centre for ADHD and Neurodevelopmental Disorders Across the Lifespan, Institute of Mental Health, Division of Psychiatry and Applied Psychology, School of Medicine, University of Nottingham, Nottingham, UK; b Department of Medical Epidemiology and Biostatistics, Karolinska Instituet, Stockholm, Sweden; c Department of Applied Health Science, School of Public Health, Indiana University, Bloomington, IN, USA; d Department of Psychological and Brain Sciences, College of Arts and Sciences, Indiana University, Bloomington, IN, USA; e Department of Psychiatry and Neurochemistry, Institute of Neuroscience and Physiology, University of Gothenburg, Gothenburg, Sweden; f School of Medical Sciences, Örebro University, Örebro, Sweden

## Abstract

**Background:**

There is a clinical concern that prescribing methylphenidate, the most common pharmacological treatment for attention-deficit hyperactivity disorder (ADHD), might increase the risk of psychotic events, particularly in young people with a history of psychosis. We aimed to determine whether the risk of psychotic events increases immediately after initiation of methylphenidate treatment or, in the longer term, 1 year after treatment initiation in adolescents and young adults with and without a previously diagnosed psychotic disorder.

**Methods:**

In this cohort study, we used population-based observational data from the Swedish Prescribed Drug Register, the National Patient Register, and the Total Population Register, three population-based registers containing data on all individuals in Sweden, to attain data on sex, birth, death, migration, medication use, and psychotic events for all eligible participants. We screened individuals on these registers to identify those receiving methylphenidate treatment, and who were aged 12–30 years at the start of treatment, for their inclusion in the study. We used a within-individual design to compare the incidence of psychotic events in these individuals during the 12-week periods immediately before and after methylphenidate initiation. Longer term risk was assessed by comparing the incidence of psychotic events 12 weeks before methylphenidate initiation and during a 12-week period one calendar year before the initiation of methylphenidate with the incidence of these events during the 12-week period one calendar year after methylphenidate initiation. We estimated the incidence rate ratios (IRR) and 95% CIs of psychotic events after the initation of methylphenidate treatment, relative to the events before treatment, which were defined as any hospital visit (inpatient admission or outpatient attendance, based on data from the National Patient Register) because of psychosis, using the International Classification of Diseases version 10 definition. Analyses were stratified by whether the individual had a history of psychosis.

**Findings:**

We searched the Swedish Prescribed Drug Register to find eligible individuals who had received methylphenidate between Jan 1, 2007 and June 30, 2012. 61 814 individuals were screened, of whom 23 898 (38·7%) individuals were assessed and 37 916 (61·3%) were excluded from the study because they were outside of the age criteria at the start of treatment, they had immigrated, emigrated, or died during the study period, or because they were administered other ADHD medications. The median age at methylphenidate initiation was 17 years, and a history of psychosis was reported in 479 (2·0%) participants. The IRR of psychotic events in the 12-week period after initiation of methylphenidate treatment relative to that in the 12-week period before treatment start was 1·04 (95% CI 0·80–1·34) in adolescents and young adults without a history of psychosis and 0·95 (0·69–1·30) among those with a history of psychosis.

**Interpretation:**

Contrary to clinical concerns, we found no evidence that initiation of methylphenidate treatment increases the risk of psychotic events in adolescents and young adults, including in those individuals with a history of psychosis. Our study should reassure clinicians considering initiating methylphenidate treatment for ADHD in adolescents and young adults, and it challenges the widely held view in clinical practice that methylphenidate should be avoided, or its use restricted, in individuals with a history of psychosis.

**Funding:**

Swedish Research Council, National Institute of Mental Health, UK National Institute of Health Research Nottingham Biomedical Research Centre.

## Introduction

Longitudinal studies^1^, ^2^ have shown that attention-deficit hyperactivity disorder (ADHD) in childhood is a risk factor for a diagnosis of psychosis in adult life. Research indicates that these disorders share common genetic^3^ and environmental aetiologies.^1^, ^4^ A potential mediator of the association between ADHD and psychosis is the prescription of central stimulants for ADHD, which causes considerable concern for clinicians.^5^, ^6^ Clinical guidelines^7^ recommend stimulant medications, including methylphenidate and dexamphetamine, as first-line treatments for ADHD. Central stimulants act as indirect dopamine agonists and are presumed to amplify neuronal signalling by prompting a marked increase in the extracellular concentration of neurotransmitters in the prefrontal cortex of the brain.^8^ Increased concentrations of synaptic dopamine have also been implicated in the generation of psychotic symptoms.^9^ Hence, the pharmacological mechanism of central stimulant medication can be viewed by clinicians as having the potential to induce psychotic symptoms and disorders.^10^

**Research in context**

**Evidence before this study**

Clinical concerns have been raised that prescribing methylphenidate, the most common pharmacological treatment for attention-deficit hyperactivity disorder (ADHD), might increase the risk of treatment-emergent psychotic events, particularly in young people with a history of psychosis. This perceived risk can lead to clinicians withholding methylphenidate treatment or using less effective alternatives. We searched PubMed for studies published in English on or after Jan 1, 2000, that investigated the association between methylphenidate prescribing, ADHD, and psychosis. We screened abstracts for relevance by use of combinations of the search terms “methylphenidate OR stimulants”, “ADHD”, and “psychosis”. We also reviewed clinical guidelines from national bodies, including the UK National Institute of Health and Care Excellence and relevant Cochrane systematic reviews. There is no clear consensus as to whether methylphenidate treatment, particularly in older adolescents and young adults and in individuals with a history of psychosis, increases the risk of psychotic events. Some observational studies that report an increased risk of psychotic events associated with methylphenidate might be affected by confounding by indication. Clinical trial data is also relatively unhelpful in answering this question because of the routine exclusion of participants with previous psychosis, short follow-up periods, and the low incidence of psychotic events in these studies.

**Added value of this study**

We examined the risk of psychotic events in more than 23 000 adolescents and young adults after initiation of methylphenidate medication by use of a within-individual comparison design that controls for confounding by indication. Contrary to clinical concerns, we did not detect an increased risk of psychotic events in adolescents and young adults after starting methylphenidate treatment. Notably, this finding also applies to individuals with a history of psychosis. We did not study other, less frequently used ADHD medications, such as atomoxetine and amphetamines (eg, dexamphetamine) and, hence, we cannot generalise our findings to these medications.

**Implications of all the available evidence**

Our study challenges the widely held view that methylphenidate should be avoided or its use restricted in individuals with a history of psychosis. It is important to confirm our findings regarding the immediate risk of psychotic events following methylphenidate initiation in other sample populations and with other study designs, which should include examining the effect of concomitant antipsychotic medication use in those with a history of psychosis.

Data from the UK Medicines and Healthcare Products Agency's Yellow Card scheme showed that, of 1335 adverse drug reaction reports regarding methylphenidate that had been received by the end of July, 2015, 663 adverse reactions were associated with psychiatric disorders, making these disorders the most frequently reported class of adverse drug reactions.^11^ Among these reports, 105 (15·8%) patients reported hallucinations, psychosis or psychotic disorders, or schizoaffective disorders. Moreover, in a US Food and Drug Administration review^12^ of data from 49 randomised controlled clinical trials investigating the effects of central stimulant medication in children, 11 adverse events related to psychosis or mania were observed during 743 person-years of follow-up in 5717 individuals (1·48 events per 100 person-years, or one event in every 70 years of treatment), versus no events reported with placebo, giving a number needed to harm of 526 patients.

Given these reports of treatment-emergent psychotic events with central stimulant medication, clinicians have been concerned that methylphenidate and other psychostimulants might provoke psychosis.^5^ Some clinicians even consider the use of stimulant ADHD medication to be contraindicated in patients with a history of psychosis.^6^ The clinical challenge of managing the potential risk of central stimulant treatment-emergent psychosis in patients with ADHD, and especially in those patients with history of a psychosis, has become more pressing with the increasing recognition, diagnosis, and treatment of ADHD in later adolescence and early adult life.^7^ Clinicians face a therapeutic dilemma without clear evidence to guide them when balancing the potential risk of psychotic events with the benefits of stimulants that are the first-line treatment for ADHD in adolescent and adult patients.^7^, ^13^ Therapeutic uncertainty is greatest in patients with a history of psychosis who, theoretically, are assumed to be at greatest risk of stimulant treatment-emergent psychotic events^10^ but who are also typically excluded from randomised clinical trials of ADHD medication.

Some observational studies^6^, ^14^ that report an increased risk of psychotic events associated with methylphenidate might be affected by confounding by indication; that is, patients who receive stimulant medication for ADHD are inherently different from those who do not and could have a greater risk of psychotic events independently of stimulant prescription. In a 2016 study that adjusted for confounding by indication, Man and colleagues^15^ used a within-individual case series design in a population of children and adolescents in Hong Kong, and they found no increased risk of psychotic events during methylphenidate treatment.

However, a Cochrane systematic review^16^, ^17^ of ten randomised controlled trials (comprising 1103 participants) and 17 non-randomised studies (comprising 76 237 participants) was unable to confirm or refute whether methylphenidate increases the risk of psychotic symptoms in children and adolescents with ADHD.

The aim of our pharmacovigilance study was twofold: first, we aimed to examine the risk of psychotic events immediately after initiation of methylphenidate treatment in adolescents and young adults with and without a previously diagnosed psychotic disorder. Second, we aimed to examine the longer term outcomes of methylphenidate exposure 1 year after treatment initiation on the risk of psychosis.

## Methods

### Study design and participants

**Figure 1:**
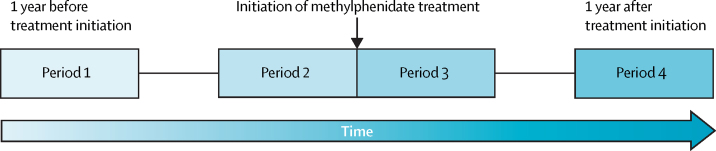
Study design Period 1: the same 12 calendar weeks as period 2, 1 year before treatment. Period 2: 12 weeks before treatment initiation. Period 3: 12 weeks after treatment initiation. Period 4: the same 12 calendar weeks as period 2, 1 year after treatment.

In this population-based cohort study, we adopted a within-individual study design to compare the incidence of psychotic events within the same patients by comparing the incidence of events during four 12-week observation periods: two periods of 12 weeks, before and after methylphenidate initiation; the period of 12 weeks one calendar year before methylphenidate initiation; and the period of 12 weeks one calendar year after methylphenidate initiation (figure 1).

Our study was based in Sweden, and we used three population-based registers to access data from all eligible individuals in the country. The study population comprised new users of methylphenidate who were aged 12–30 years at methylphenidate treatment initiation. New users were defined as individuals with a first registered dispensation of methylphenidate (Anatomical Therapeutic Chemical [ATC] code N06BA04) between Jan 1, 2007, and June 30, 2012, who were identified by use of the Swedish Prescribed Drug Register. New users included those who had initiated methylphenidate treatment for the first time and those who had not received methylphenidate for at least 1·5 years, since the Swedish Prescribed Drug Register began on July 1, 2005. We excluded individuals from the study if they had received ADHD medications other than methylphenidate during the study period, they had immigrated into Sweden after the start of period 1 (ie, one calendar year before treatment initiation), or if they had died or emigrated before the end of period 4 (ie, one calendar year after treatment initiation).

The study was approved by the Regional Ethics Committee at Karolinska Instituet (Stockholm, Sweden). All data were pseudonymised before linkage and analysis, and individual records were completely unidentifiable during the analysis. Since our study was based on population registers, the requirement for informed consent was waived.

### Data sources

We used the participants' personal identification numbers,^18^ which serve as unique identifiers for all residents, to access patient data in three population-based registers in Sweden; these data include details on medication use (to enable assessment for study inclusion and of methylphenidate treatment) and psychotic events (to assess outcomes). The Swedish Prescribed Drug Register^19^ contains data on all drugs that have been dispensed in Sweden since July, 2005, including dispensation dates and active ingredients of the drugs, which are coded according to the ATC Classification System. The National Patient Register^20^ includes information on psychiatric inpatient care since 1973 and outpatient visits to specialists since 2001. Each contact with specialty care is recorded and includes information on one primary and up to eight secondary diagnoses, which are coded according to the tenth revision of the International Classification of Diseases (ICD-10) from 1997 onwards. Most diagnoses in the register have a positive predictive value of about 85–95%.^20^ The Total Population Register^21^ provides information on sex, birth, death, and migration for all individuals in Sweden.

### Outcomes

Psychotic events were defined as any hospital visits (inpatient admission or outpatient attendance) because of psychosis, according to tvhe recorded ICD-10 codes (F1x.5x, F06·0, F06·2, F23, F28, F29, F30·2, F31·2, F31·5, F32·3, F33·3, R440, R442, and R443), as recorded in the National Patient Register. Chronic psychotic conditions were also included (ICD-10 codes F20, F22, and F25). We performed all analyses separately in patients with and without a history of psychosis.

To investigate the immediate risk of psychotic events after initiation of methylphenidate treatment, we compared the incidence of these events during the two periods of 12 weeks on either side of methylphenidate initiation (figure 1), designated period 2 (the 12-week period before treatment start) and period 3 (the 12-week period after treatment start) in those meeting inclusion and exclusion criteria. To assess the longer term risk of methylphenidate, we compared the incidence of psychotic events during the 12-week period immediately before methylphenidate initiation (period 2) with that during the 12-week period one calendar year after methylphenidate initiation (designated period 4; ie, approximately 52 weeks to 64 weeks after treatment). These represented the primary analyses.

In a secondary analysis, we compared the incidence of psychotic events during the 12-week period one calendar year after methylphenidate initiation (period 4) with that during the 12-week period one calendar year before methylphenidate initiation (period 1; ie, approximately 64 weeks to 52 weeks before treatment; figure 1) in those meeting inclusion and exclusion criteria. For completeness, we provided results from the comparisons between periods 2, 3, and 4 and period 1 (reference).

As prespecified analyses, we also stratified the main analyses by age of incident methylphenidate use (adolescent, 12–17 years *vs* young adult, 18–30 years), to investigate how the incidence of psychotic events differed by age at treatment initiation. Patients with at least one psychotic event before period 1 were considered to have had a history of psychosis. Finally, we repeated the analyses after excluding patients with chronic psychotic conditions, such as schizophrenia (F20.x), persistent delusional disorders (F22.x), and schizoaffective disorders (F25.x).

### Statistical analysis

We used conditional Poisson regression models to compare the incidence of psychotic events during a potential risk period (period 2, 3, or 4) and the incidence during a selected baseline period (period 2 [primary analysis] or period 1 [secondary analysis]), with each patient as a separate stratum. Only those patients who had at least one psychotic event during the selected time periods contributed data to the analysis. The results are presented as incidence rate ratios (IRRs) and 95% CIs.

In within-individual comparisons, since each patient served as his or her own control, all time-constant factors that vary between patients (such as genetics and baseline disease severity) were automatically adjusted for. Moreover, seasonal effects were also adjusted for, since the 12-week period after treatment was compared with equivalent 12-week periods in the previous and subsequent years. To test the robustness of results from a 12-week period, we also did sensitivity analyses by setting the length of each observation period to be 8 and 24 weeks, instead of 12 weeks. As for the main analyses, all time-constant factors were adjusted for by comparing the same individual with themselves. No data were missing.

All tests of statistical hypotheses were assessed at a two-sided 5% level of significance. Data management was done with SAS version 9.4, and R software version 3.4.3 was used for statistical analyses.

### Role of the funding source

The funder of the study had no role in study design, data collection, data analysis, data interpretation, or writing of the report. The corresponding author had full access to all the data in the study and had final responsibility for the decision to submit for publication.

## Results

We searched the Swedish Prescribed Drug Register to find individuals who had received methylphenidate between Jan 1, 2007 and June 30, 2012. We identified 61 814 individuals who had started treatment with methylphenidate. However, we excluded 30 614 (49·5%) individuals because they did not meet age criteria, 400 (0·6%) individuals because they had emigrated, immigrated, or died during the study period, and 6902 (11·2%) individuals because they had received other ADHD medications, giving a study population of 23 898 (38·7%) individuals (appendix p 5).

The cohort included 9729 (40·7%) women and 14 169 (59·3%) men (table 1). The median age at methylphenidate initiation was 17 years (IQR 14–22). A history of psychosis was observed in 479 (2·0%) participants. 304 (1·3%) individuals had at least one psychotic event during the study period. The overall incidence of psychotic events was 5·64 events per 10 000 person-weeks (95% CI 5·38–5·91); in those with a history of psychosis, the incidence was 113·76 events per 10 000 person-weeks (105·61–122·53) and, in those without a history of psychosis, the incidence was 3·43 events per 10 000 person-weeks (3·23–3·65).

**Table 1:** Study population characteristics

		**Characteristics (n=23898)**
Sex, n (%)		
	Female	9729 (40·7%)
	Male	14169 (59·3%)
Median age at treatment initiation (IQR), years		17 (14–22)
Individuals with at least one psychotic event, n (%)		
	Period 1	89 (0·4%)
	Period 2	114 (0·5%)
	Period 3	110 (0·5%)
	Period 4	94 (0·4%)

Period 1: the 12-week period one calendar year before treatment. Period 2: 12 weeks before treatment initiation. Period 3: 12 weeks after treatment initiation. Period 4: the 12-week period one calendar year after treatment.

When we compared the incidence of these events during the 12-week periods before and after initiation of methylphenidate treatment (period 3 *vs* period 2), we found no difference in the immediate risk of psychotic events after treatment initiation in individuals with a history of psychosis (IRR 0·95, 95% CI 0·69–1·30; table 2). Similarly, among those without a history of psychosis, we found no difference in the immediate risk of psychotic events after initiation of methylphenidate treatment (1·04, 0·80–1·34). However, relative to the period immediately before initiation of methylphenidate treatment (period 2), the IRR of psychotic events 1 year later (period 4) was reduced by 36% in those with a history of psychosis (IRR 0·64, 95% CI 0·45–0·91; table 2). In those without a history of psychosis, the IRR was reduced by 18% (0·82, 0·62–1·07; period 2 *vs* period 4), but this difference was not significant.

**Table 2:** Short-term and long-term risk of psychotic events after methylphenidate treatment initiation

	**Participants with at least one event**	**Number of events**	**Person-weeks**	**Incidence per 10000 person-weeks (95% CI)**	**Primary analysis IRR (95% CI)**	**Secondary analysis IRR (95% CI)**
**Individuals with a history of psychosis (n=479)**						
Period 1	55 (11·5%)	98	5748	170·49 (139·87–207·82)	··	1 (ref)
Period 2	44 (9·2%)	78	5748	135·70 (108·69–169·42)	1 (ref)	0·80 (0·59–1·07)
Period 3	45 (9·4%)	74	5748	128·74 (102·51–161·68)	0·95 (0·69–1·30)	0·76 (0·56–1·02)
Period 4	30 (6·3%)	50	5748	86·99 (65·93–114·77)	0·64 (0·45–0·91)	0·51 (0·36–0·72)
**Individuals without a history of psychosis (n=23419)**						
Period 1	34 (0·1%)	54	281028	1·92 (1·47–2·51)	··	1 (ref)
Period 2	70 (0·3%)	114	281028	4·06 (3·38–4·87)	1 (ref)	2·11 (1·53–2·92)
Period 3	65 (0·3%)	118	281028	4·20 (3·51–5·03)	1·04 (0·80–1·34)	2·19 (1·58–3·02)
Period 4	64 (0·3%)	93	281028	3·31 (2·70–4·06)	0·82 (0·62–1·07)	1·72 (1·23–2·41)

Data are for the whole cohort (aged 12–30 years at the start of methylphenidate treatment). Period 1: the 12-week period one calendar year before treatment. Period 2: 12 weeks before treatment initiation. Period 3: 12 weeks after treatment initiation. Period 4: the 12-week period one calendar year after treatment. The primary analysis IRR compares periods 2 and 4; the secondary analysis IRR compares all periods with period 1. IRR=incidence rate ratio of psychotic events.

**Figure 2:**
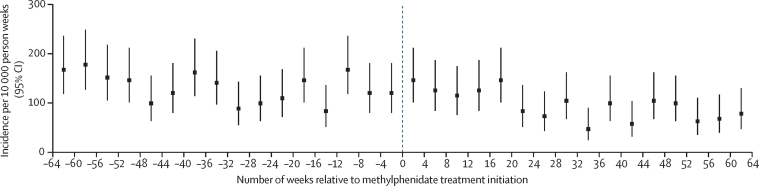
Incidence of hospital visits because of psychosis per 10 000 person-weeks, during the 64 weeks before and after methylphenidate treatment initiation in individuals with a history of psychosis

**Figure 3:**
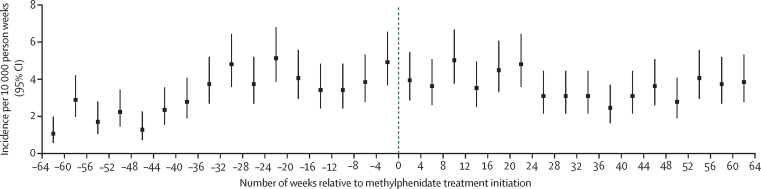
Incidence of hospital visits because of psychosis per 10 000 person-weeks, during the 64 weeks before and after methylphenidate treatment initiation in individuals without a history of psychosis

The incidence of hospital visits attributed to psychotic events during the 64 weeks before and after methylphenidate medication are shown in figure 2 (for those with a history of psychosis) and figure 3 (for those without). The incidence of psychotic events appeared to be similar before initiation of methylphenidate treatment as it was after initiation of treatment (in individuals with and those without a history of psychosis). In all individuals, the incidence of events decreased during the year after initiation of methylphenidate treatment.

In a secondary analysis to compare the incidence of events in period 4 (1 year after treatment start) relative to period 1 (1 year before treatment start), the IRR of psychotic events in those with a history of psychosis was reduced by 49% (IRR 0·51, 95% CI 0·36–0·72; table 2). In those without a history of psychosis, the IRR of psychotic events in period 4 versus period 1 was increased by 72% (1·72, 1·23–2·41). However, with period 1 as the baseline reference, there was a similar risk of psychotic events both immediately before (period 2 *vs* period 1: 2·11, 1·53–2·92) and immediately after initiation of methylphenidate treatment (period 3 *vs* period 1: 2·19, 1·58–3·02) in those without a history of psychosis.

When stratifying by age group, we found no difference in the immediate risk of psychotic events (ie, comparing period 3 *vs* period 2) in adolescents (aged 12–17 years; IRR 0·42, 95% CI 0·23–0·79; appendix p 1). We also found no difference in the longer term risk of psychosis (ie, period 4 *vs* period 2) in adolescents (0·88, 0·53–1·45). There were too few events in adolescents with a history of psychosis to draw robust conclusions about the differences between those with and those without a history of psychosis. We also found no difference in the immediate risk of psychotic events (period 3 *vs* period 2) in young adults (age 18–30 years) with a history of psychosis (0·96, 0·70–1·32) nor in those without (1·27, 0·95–1·69; appendix p 2). Finally, we found no difference in the longer term risk of psychosis (period 4 *vs* period 2) in young adults with a history of psychosis (0·57, 0·39–0·82) nor those without (0·86, 0·62–1·17).

When we excluded participants with chronic psychotic conditions (diagnoses F20.x, F22.x or F25.x; n=268) from analyses (appendix p 3), we found similar trends to those seen in the primary and secondary analyses (table 2). When we used the incidences of events during the immediate period before initiation of methylphenidate treatment (period 2) as the reference values, we found no difference in the IRR of psychotic events immediately after initiation of methylphenidate treatment when excluding those with chronic psychotic diagnoses (IRR 0·98, 95% CI 0·78–1·24). However, relative to period 2, the IRR of psychotic events 1 year after the start of treatment (period 4) was reduced by 29% (0·71, 0·55–0·91). Finally, all results were similar to those of the primary and secondary analyses when the observation period was varied to 8 weeks or 24 weeks (appendix p 4).

## Discussion

We examined the risk of psychotic events in more than 23 000 adolescents and young adults after initiation of methylphenidate treatment by use of a within-individual comparison design. Notably, we found no evidence of an increase in the immediate risk of psychotic events when comparing the 12-week periods before and after initiation of methylphenidate treatment, neither for those with or without a history of psychosis. However, 1 year after the start of methylphenidate treatment, we observed a 36% reduction in the incidence of psychotic events in those with a history of psychosis and an 18% reduction in incidence in those without a history of psychosis relative to the period immediately before initiation of methylphenidate treatment. This finding challenges the widely held view that methylphenidate should be avoided or its use restricted in individuals with a history of psychosis.^6^, ^22^

In a secondary analysis of the longer term risks, we found no difference in the risk of psychotic events in those with a history of psychosis when comparing the incidence of events during the 12-week period 1 year after methylphenidate initiation with that during the 12-week period 1 year before the initiation of methylphenidate. In those with no history of psychosis, the incidence of psychotic events was greater immediately before and after initiation of methylphenidate treatment and 1 year after initiation of methylphenidate treatment relative to that 1 year before treatment started.

When we observed the incidence of psychotic events over an extended period of 64 weeks before and 64 weeks after the initiation of methylphenidate treatment, two different trends emerged. First, in those with a history of psychosis, the overall incidence of psychotic events decreased over time, but the incidence was similar immediately before and after initiation of methylphenidate treatment. This finding could be explained by a regression to the mean, since we would expect that, in any population selected for high severity (ie, previous psychotic events), there would be a decline in the incidence of these events after the initial selection point. Second, in those without a history of psychosis, the overall incidence increased over time, but the incidence was similar immediately before and after initiation of methylphenidate. Given that the proposed pharmacological mechanism linking stimulant medication and psychotic symptoms predicts an immediate post-exposure effect rather than a longer term effect, the increasing incidence with time could be explained by an age effect, with the background incidence of psychotic events increasing during a 2-year observation period for individuals aged 12–30 years.

We found a similar incidence of psychotic events before as after methylphenidate initiation, in agreement with Man and colleagues,^15^ which suggests that methylphenidate initiation might be driven by factors related to the emergence of psychosis rather than methylphenidate triggering psychotic events. Contrary to previous reports^6^ and clinical guidelines,^22^ we found no evidence to suggest that the risk of psychosis associated with methylphenidate was greater in those with a history of psychosis. With a similar within-individual design, Viktorin and colleagues^23^ observed an increased risk of mania (which might include mania with psychotic symptoms: F30·1 and F31·1) after methylphenidate monotherapy in a smaller cohort of 2307 adults with bipolar disorder and comorbid ADHD. Hence, our findings do not exclude the risk that methylphenidate might elicit a manic episode with psychotic symptoms in bipolar disorder patients who are not receiving mood stabilisers.

The strengths of our study include the large number of participants and the naturalistic clinical setting, in which we used validated clinical diagnoses.^20^ Unlike previous studies,^5^, ^15^ we investigated the longer term outcome of methylphenidate exposure 1 year after treatment initiation. Our study includes individuals who, for various reasons, would have been excluded from or would not have volunteered for a clinical trial and provides a longer observation period than most trials. Moreover, the use of a within-individual design automatically adjusts for many individual-specific factors, including ADHD severity, history of and genetic susceptibility to psychosis, and environmental factors, which would not be possible to adjust for in a conventional epidemiological approach. This design provides an improved strategy for handling selection effects and confounding by indication in pharmaco-epidemiological studies.

A limitation of our study is that, although the design allows for extensive adjustment of otherwise unmeasured confounders, it does not adjust for changes that might have occurred during the 24-month observation period. Given the observational nature of the study, we cannot rule out the possibility that unmeasured factors might be specific to each group. Hence, the increasing incidence of psychotic events over 24 months in participants without a history of psychosis might be attributable to an age effect on the incidence of psychotic diagnoses rather than the effect of methylphenidate treatment. However, it could also reflect further unmeasured exposure to methylphenidate. It is thus important to confirm the findings regarding the immediate risk of psychotic events after initiation of methylphenidate in other samples and with other study designs.

Further limitations of our study include an absence of information on methylphenidate dosage, missed doses, and the possibility that milder psychotic events not resulting in hospital visits might have been missed. Our pharmacovigilance study of methylphenidate was limited to psychotic events, and we did not consider other adverse effects or therapeutic benefits.^17^, ^24^ We included the coding of drug-induced psychosis to ensure that stimulant-related psychotic adverse events were recorded. We did not select participants because of their ADHD diagnosis, although this is the only clinically indicated use for methylphenidate. We studied clinically prescribed methylphenidate use, and so we cannot generalise our findings to non-clinically prescribed use. We cannot exclude the possible moderating effect of comorbid conditions on the effects of methylphenidate. Additionally, we did not study other, less frequently used, ADHD medications such as atomoxetine and amphetamines (eg, dexamphetamine) and, hence, we cannot generalise our findings to these medications, which could carry different risk.^25^ Finally, we focused on psychotic events associated with methylphenidate treatment, but we did not assess concomitant mediation use, including use of antipsychotics. Hence, we cannot determine from our study whether the risk of psychotic events associated with methylphenidate in individuals with a history of psychosis is moderated by antipsychotic medication.

In conclusion, contrary to clinical concerns, our study did not detect an increased risk of psychotic events in adolescents and young adults after starting methylphenidate treatment. Notably, this finding also applies to individuals with a history of psychosis.
