# Supplementary material for: Methylphenidate and the risk of psychosis in adolescents and young adults: a population-based cohort study
Source: Lancet Psychiatry. 2019 Aug;6(8):651–8. doi: 10.1016/S2215-0366(19)30189-0 (PMC6646837; doi:10.1016/S2215-0366(19)30189-0)

THE LANCET
Psychiatry

Supplementary appendix

This appendix formed part of the original submission and has been peer reviewed. We post it as supplied by the authors.

Supplement to: Hollis C, Chen Q, Chang Z, et al. Methylphenidate and the risk of psychosis in adolescents and young adults: a population-based cohort study. *Lancet Psychiatry* 2019; published online June 17. http://dx.doi.org/10.1016/S2215-0366(19)30189-0.

SUPPLEMENTARY MATERIAL

TABLE 3: Adolescents age 12 to 17 years: Short and long-term risk of psychotic events following methylphenidate treatment initiation

Observational period	Individuals with at least one event, No.	Events, No.	Person-weeks	Incidence rate (95% CI) per 10,000 person-weeks	Primary analysis IRR (95% CI)	Secondary analysis IRR (95% CI)
Period 1	10	20	157,212	1.27(0.82 to 1.97)	--	Reference
Period 2	18	33	157,212	2.10(1.49 to 2.95)	Reference	1.65(0.95 to 2.88)
Period 3	12	14	157,212	0.89(0.53 to 1.50)	0.42(0.23 to 0.79)	0.70(0.35 to 1.39)
Period 4	16	29	157,212	1.84(1.28 to 2.65)	0.88(0.53 to 1.45)	1.45(0.82 to 2.56)

IRR: incidence rate ratio of psychotic events; CI confidence interval

TABLE 4: Young adults age 18 to 30: Short and long-term risk of psychotic events following methylphenidate treatment initiation

Observational period	Individuals with at least one event, No.	Events, No.	Person-weeks	Incidence rate (95% CI) per 10,000 person-weeks	Primary analysis<br><br>IRR (95% CI)	Secondary analysis<br><br>IRR (95% CI)
With prior psychosis						
Period 1	49	85	5352	158.82(128.40 to 196.44)	--	Reference
Period 2	43	76	5352	142.00(113.41 to 177.80)	Reference	0.89(0.66 to 1.22)
Period 3	44	73	5352	136.40(108.44 to 171.57)	0.96(0.70 to 1.32)	0.86(0.63 to 1.17)
Period 4	28	43	5352	80.34(59.59 to 108.33)	0.57(0.39 to 0.82)	0.51(0.35 to 0.73)
Without prior psychosis						
Period 1	30	47	124,212	3.78(2.84 to 5.04)	--	Reference
Period 2	53	83	124,212	6.68(5.39 to 8.29)	Reference	1.77(1.23 to 2.53)
Period 3	54	105	124,212	8.45(6.98 to 10.24)	1.27(0.95 to 1.69)	2.23(1.58 to 3.15)
Period 4	50	71	124,212	5.72(4.53 to 7.21)	0.86(0.62 to 1.17)	1.51(1.04 to 2.18)

IRR: incidence rate ratio of psychotic events; CI confidence interval

Table 5. Risk of psychotic events for whole sample after excluding individuals with long-term diagnoses (F20.x, F22.x, and F25.x)

Incidence rates of hospital visits due to psychosis during selected observational periods and incidence rate ratios

**Observational period**	**Events, No.**	**Person-weeks**	**Incidence rate per 10,000 person-weeks (95% CI)**	**Primary Analysis IRR (95% CI)**	**Secondary Analysis IRR (95% CI)**
Period 1	103	283,560	3.63(2.99 to 4.41)	--	Reference
Period 2	143	283,560	5.04(4.28 to 5.94)	Reference	1.39(1.08 to 1.79)
Period 3	140	283,560	4.94(4.18 to 5.83)	0.98(0.78 to 1.24)	1.36(1.05 to 1.75)
Period 4	101	283,560	3.56(2.93 to 4.33)	0.71(0.55 to 0.91)	0.98(0.75 to 1.29)

IRR: incidence rate ratio of psychotic events; CI confidence interval

Table 6. Risk of psychotic events for whole sample with different observation periods of 8, 12 and 24 weeks

Incidence rates of hospital visits due to psychosis during selected observational periods and incidence rate ratios

Observational period	Events, No.	Person-weeks	Incidence rate per 10,000 person-weeks (95% CI)	Primary Analysis IRR (95% CI)	Secondary Analysis IRR (95% CI)
n = 8, weeks					
Period 1	112	191,256	5.86(4.87 to 7.05)	--	Reference
Period 2	128	191,256	6.69(5.63 to 7.96)	Reference	1.14(0.89 to 1.47)
Period 3	123	191,256	6.43(5.39 to 7.67)	0.96(0.75 to 1.23)	1.10(0.85 to 1.42)
Period 4	98	191,256	5.12(4.20 to 6.25)	0.77(0.59 to 1.00)	0.88(0.67 to 1.15)
n = 12, weeks					
Period 1	152	286,776	5.30(4.52 to 6.21)	--	Reference
Period 2	192	286,776	6.70(5.81 to 7.71)	Reference	1.26(1.02 to 1.56)
Period 3	192	286,776	6.70(5.81 to 7.71)	1.00(0.82 to 1.22)	1.26(1.02 to 1.56)
Period 4	143	286,776	4.99(4.23 to 5.87)	0.74(0.60 to 0.92)	0.94(0.75 to 1.18)
n = 24, weeks					
Period 1	265	572,784	4.63(4.10 to 5.22)	--	Reference
Period 2	375	572,784	6.55(5.92 to 7.24)	Reference	1.42(1.21 to 1.66)
Period 3	380	572,784	6.63(6.00 to 7.34)	1.01(0.88 to 1.17)	1.43(1.23 to 1.68)
Period 4	270	572,784	4.71(4.18 to 5.31)	0.72(0.62 to 0.84)	1.02(0.86 to 1.21)

n: length of each observational period; IRR: incidence rate ratio of psychotic events; CI confidence interval

FIGURE 4. Flow chart for study population identification

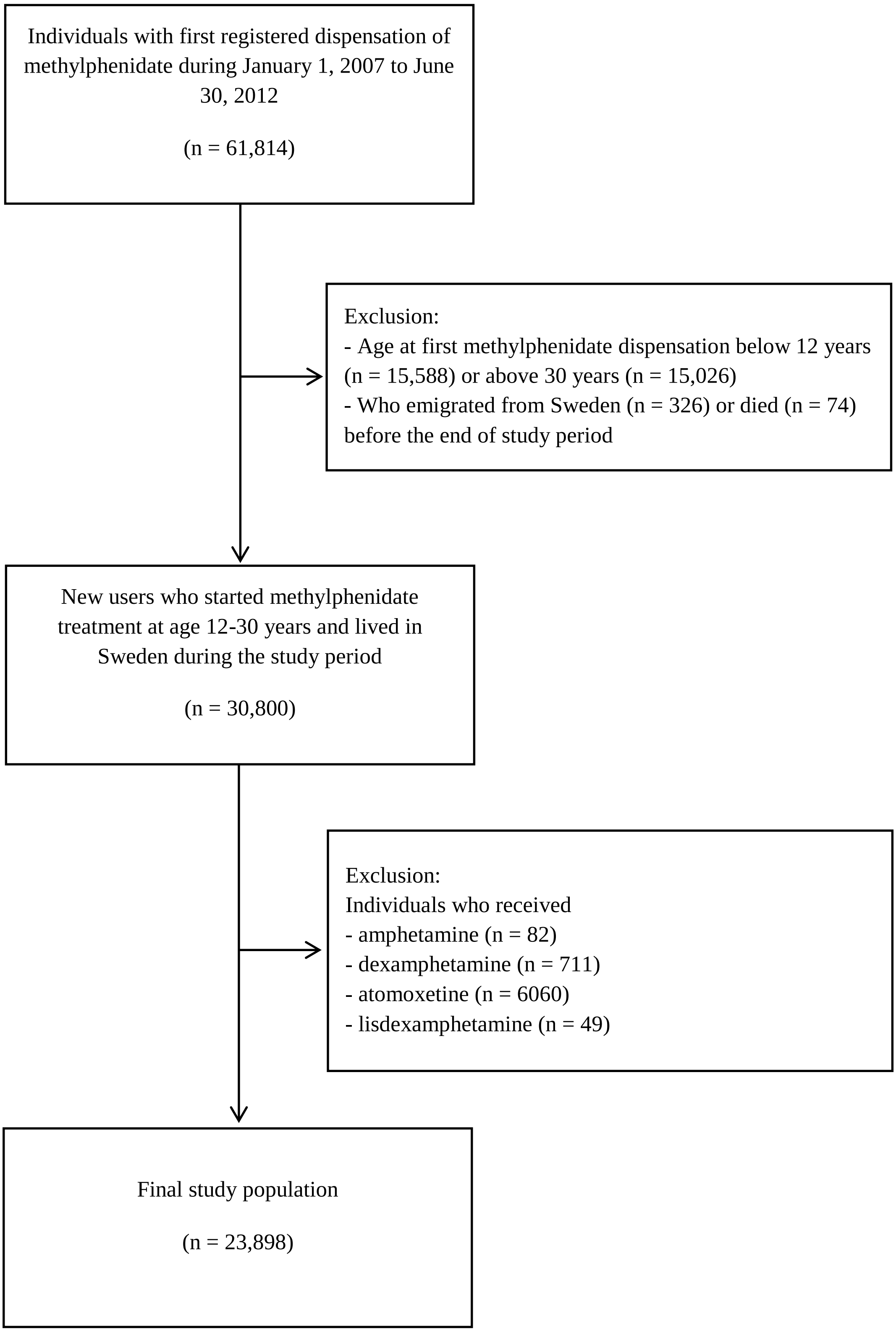

Supplement: Supplementary appendix [file mmc1.pdf]
